# Genomic characterization of Crimean-Congo hemorrhagic fever virus in *Hyalomma* ticks from Cameroon highlights circulation of the Africa 3 lineage

**DOI:** 10.1186/s13071-026-07560-5

**Published:** 2026-07-22

**Authors:** Armand Namekong Fokeng, Stéphanie Mvodo, Moréna Gasparine, Laurence Thirion, Manchang Tanyi Kinsley, Félix Fewou Mfoujouem, Rémi Charrel, Xavier de Lamballerie, Archile Paguem, Alessandra Falchi

**Affiliations:** 1 Unité des Virus Emergents (UVE: Aix-Marseille Univ, Università di Corsica, IRD 190, Inserm 1207, IRBA), Marseille, France; 2 Department of Agricultural Economics and Agribusiness, Faculty of Agriculture and Veterinary Medicine, University of Buea, Po Box: 63, SWR, Buea, Cameroon; 3 Department of Veterinary Medicine, Faculty of Agriculture and Veterinary Medicine, University of Buea, Po Box: 63 SWR, Buea, Cameroon; 4 Research Institute for Agricultural Development, Bangangté, Cameroon; 5 Centre National de Référence Des Arbovirus, Marseille, France

**Keywords:** CCHFV, Cameroon, *Hyalomma*, RT-qPCR, Nanopore sequencing, L S segments, Phylogeny, Central Africa, Zoonosis

## Abstract

**Background:**

Crimean-Congo Hemorrhagic Fever (CCHF) is a tick-borne zoonotic disease of growing public health importance worldwide. Despite its recognized presence in neighboring countries, data on the occurrence and molecular characteristics of Crimean-Congo Hemorrhagic Fever Virus (CCHFV) in ticks from Cameroon remain scarce.

**Methods:**

Between March 2023 and November 2024, ticks were collected from cattle (originating from Adamawa and North regions) at the Ngaoundéré municipal slaughterhouse located in the Adamawa region of Cameroon. Specimens were morphologically identified, pooled, and screened for CCHFV RNA using real-time RT-qPCR. Minimum infection rates (MIR) were calculated. Positive samples underwent amplification and sequencing of the S and L genomic segments, followed by phylogenetic analysis using the Maximum Likelihood method (1,000 bootstrap replicates).

**Results:**

A total of 299 adult ticks were collected from 50 cattle and grouped into 85 pools. Four pools tested positive for CCHFV RNA, all belonging to *Hyalomma rufipes* and *H. nitidum*. Partial sequences of the S (1,537 nt) and L (11,530 nt) segments were successfully obtained from one *H. rufipes* pool. Phylogenetic analysis placed both segments within genotype III (Africa 3).

**Conclusions:**

This study provides the first molecular characterization of CCHFV from *Hyalomma* ticks collected from cattle in Cameroon. The findings underscore the need to integrate molecular surveillance and tick control into national One Health strategies to prevent potential spillover to humans.

**Graphical Abstract:**

**Figure:**
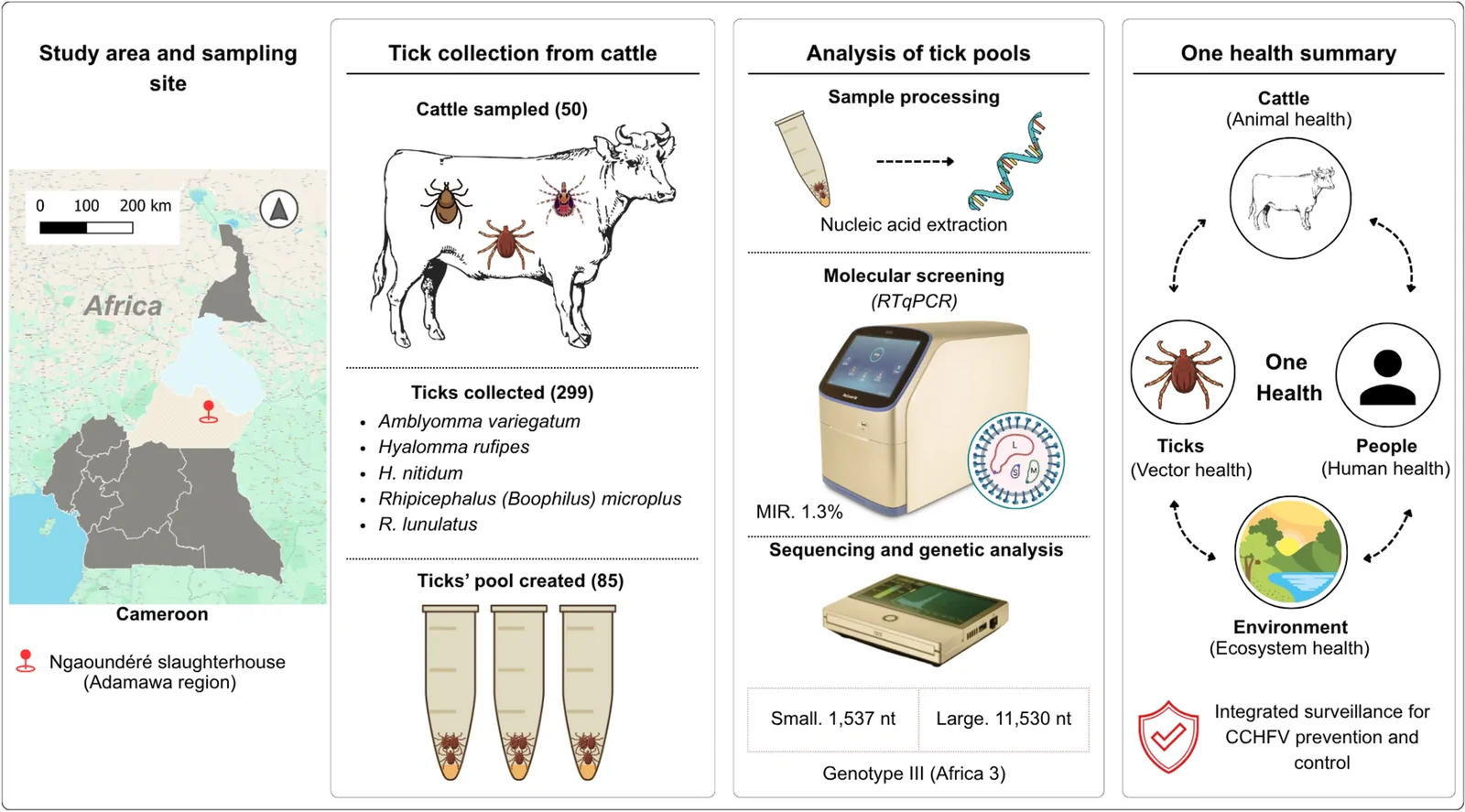

## Background

Crimean-Congo Hemorrhagic Fever (CCHF) is a tick-borne viral zoonosis caused by the Crimean-Congo Hemorrhagic Fever Virus (CCHFV), a member of the *Orthonairovirus* genus within the family *Nairoviridae*. This enveloped virus possesses a tripartite, negative-sense RNA genome comprising three segments, Small (S), Medium (M), and Large (L) which encode the nucleoprotein, the glycoprotein precursor, and the RNA-dependent RNA polymerase, respectively [1].

Crimean-Congo Hemorrhagic Fever Virus is primarily transmitted by ticks of the genus *Hyalomma*, maintaining a vertebrate–tick–vertebrate transmission cycle [2]. Wild and domestic animals, typically asymptomatic, play a critical epidemiological role by serving as amplifying hosts that facilitate viral maintenance and dissemination through infected ticks [3, 4]. In humans, the virus is mainly transmitted through bites from *Hyalomma* ticks, contact with the blood or tissues of viremic animals during slaughter, and more rarely through direct contact with the bodily fluids of an infected patient in healthcare settings. At-risk populations include livestock farmers, breeders, butchers, veterinarians, and healthcare workers [5]. Human infection with CCHFV can lead to severe hemorrhagic disease with case fatality rates ranging from 10 to 40% [6]. The absence of licensed antiviral therapies underscores the necessity of continuous surveillance of this emerging zoonotic threat in endemic regions [6].

In Cameroon, evidence of CCHFV circulation has mainly been derived from serological investigations and partial genome detections. A CCHFV seroprevalence of 56% was reported in cattle from the North and Adamawa regions [7]. Similarly, Simo Tchetgna et al. (2023) detected antibodies in 98% of adult cattle sampled in Yaoundé livestock markets, alongside molecular detection of a fragment of the L segment (445 nt) in *Hyalomma truncatum* tick pools, belonging to genotype III (Africa 3). In the Central region (Akonolinga), a One Health-based survey revealed CCHFV seroprevalences of 10.9% in cattle, 3.4% in goats, and 1.9% in rural human populations residing in close contact with domestic animals. Furthermore, two human viremia cases were confirmed by RT-qPCR and sequencing of a 150-nucleotide fragment of the L segment, also classified within the Africa 3 clade [8].

Despite this accumulating serological and molecular evidence, no extended genomic characterisation of CCHFV from tick isolates in Cameroon. This lack of genomic data limits our understanding of viral lineage diversity, evolutionary dynamics, and vector adaptation in the region. In contrast, complete sequences of the S, M and L have been obtained from human isolates in Southern Africa and Sudan, revealing reassortment events within viral lineages [9, 10]. However, such genomic insights remain geographically restricted.

In this context, the present study aimed to describe the first sequencing of the S and L segments of CCHFV obtained from ticks collected at the Ngaoundéré municipal slaughterhouse from cattle originating from two regions (Adamawa and North) of Cameroon and to determine its phylogenetic relationship for genotype assignment. These findings highlight a potential public health risk for populations in close contact with livestock.

## Methods

### Study area and collection period

**Fig. 1:**
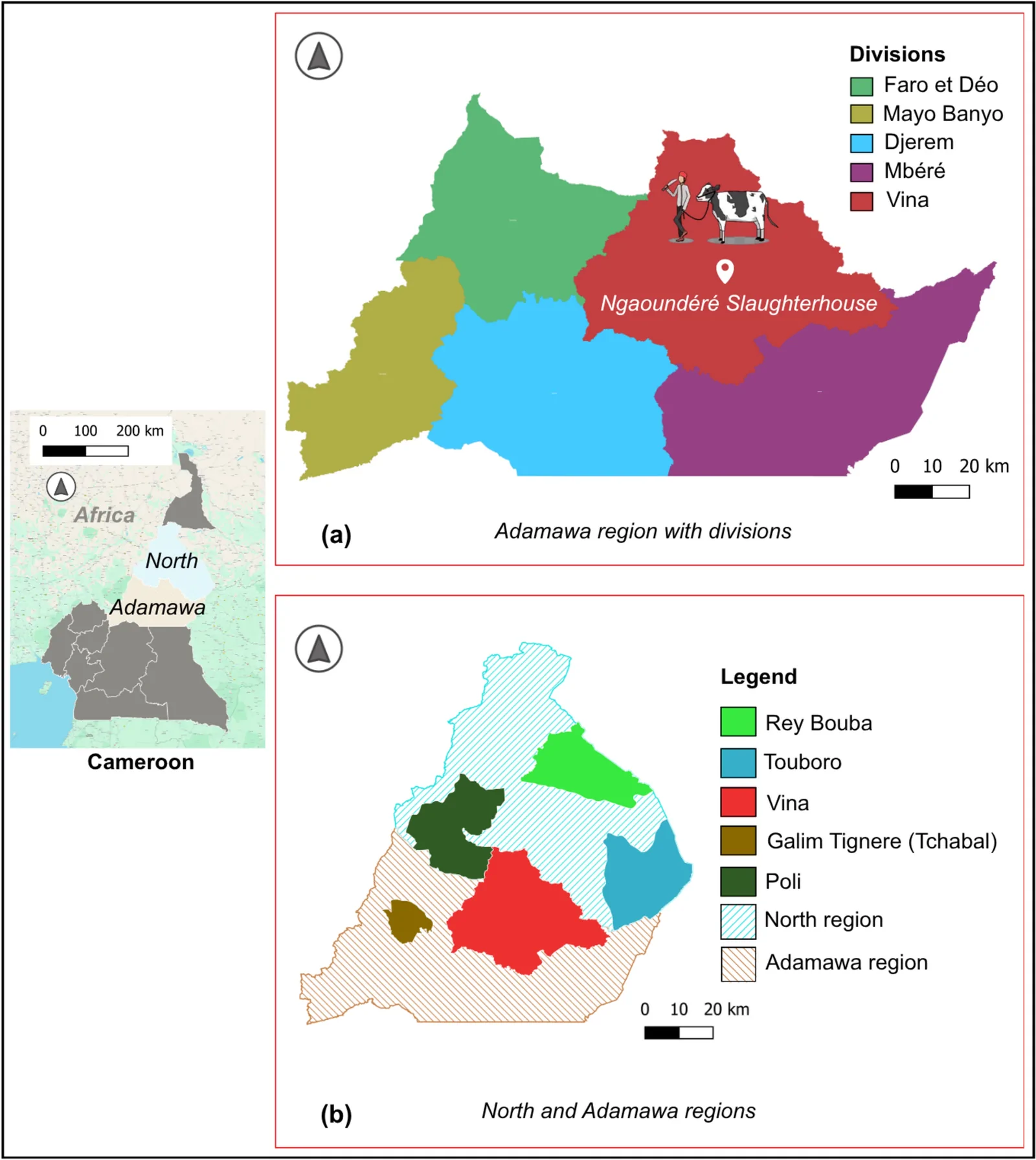
Mapping: **a** sampling site—Ngaoundéré slaughterhouse; **b** origins of the studied cattle

Sampling was conducted at the Ngaoundéré municipal slaughterhouse, located in the Adamawa Region of Cameroon, from March 2023 to November 2024. The study area (Fig. 1) lies within the Sudano-Guinean savanna agro-ecological zone and functions as a major transit hub for cattle originating from various parts of Sub-Saharan Africa [11]. The region has a tropical climate with two distinct seasons: a dry season from November to early March and a rainy season from late March or April to October [12, 13]. Cattle are the main species slaughtered at the facility, consisting primarily of Gudali, Red Fulani, and White Fulani breeds (*Bos indicus*), as well as crossbreeds with European taurine cattle. This information on breeds represents the regional context and not necessarily the characteristics of the sampled population. Animals are supplied from livestock markets located up to 400 km away, covering the Adamawa Plateau and northern Cameroon [14].

### Collection and identification of ticks

#### Sampling strategy

Sampling was conducted between March 2023 and November 2024, at the Ngaoundéré municipal slaughterhouse with a total of 24 visits (16 months for a single visit and 4 months with two visits). During each visit, approximately 15 cattle among the ~ 60 animals slaughtered per day were randomly inspected for tick infestation, resulting in approximately 360 cattle examined overall. However, only cattle infested with ticks were included in the study, leading to a final sample size of 50 animals. Among these, between 1 and 6 infested animals per visit were selected for tick collection.

Previous studies in Cameroon report a low prevalence of *Hyalomma* infestation in cattle (0.1–10%) [15, 16]. Accordingly, among approximately 60 cattle slaughtered per day at the abattoir, between 0 and 6 animals would be expected to carry *Hyalomma* ticks per visit. However, this should be interpreted as an approximate expectation rather than a precise estimate, as infestation levels may vary according to season, origin of animals, and local ecological conditions [17].

#### Collection and identification

Ticks were manually collected using fine forceps immediately after slaughter from body regions accessible during carcass processing. When *Hyalomma* ticks were detected, all co-infesting tick species present on the same animal were collected. Because animals could not be examined prior to slaughter, the inspection was limited to the carcass surfaces available at that stage.

Ticks were preserved in 70% ethanol [18] immediately after collection. All samples were considered non-infectious prior to transport and handled in accordance with biosafety regulations (ER15-2024). Inactivated samples were transported to the laboratory of the Department of Agricultural Economics and Agribusiness, Faculty of Agriculture and Veterinary Medicine, University of Buea for morphological identification using stereomicroscopy and standard taxonomic keys [19].

Transportation tubes were labelled with the animal’s identification code, age, geographical origin, and breed. Upon arrival, ticks were morphologically identified to the species level using standard dichotomous keys [19]. Specimens were then grouped into homogeneous pools (typically 1–6 ticks) according to species, developmental stage, host and sex.

### RNA extraction

Each tick pool was mechanically homogenized (using TissueLyser II, 30 Hz for 3 min, QIAGEN, Germany) in 1 mL of Minimum Essential Medium (MEM), a buffered isotonic that preserves viral stability and ensure compatibility with downstream molecular analyses. MEM medium was supplemented with antibiotics (penicillin–streptomycin and kanamycin) and an antifungal agent (amphotericin B) to prevent bacterial and fungal contamination from tick-associated microbiota or environmental sources during homogenisation and downstream molecular analyses. An internal control (MS2 bacteriophage, 10 µL per 100 µL homogenate) was added prior to RNA extraction to monitor extraction and amplification efficiency. Viral RNA was extracted from 200 µL of homogenate using the KingFisher Flex™ system (Thermo Fisher Scientific, USA) in combination with the MagMAX™ Viral/Pathogen Ultra Nucleic Acid Isolation Kit, following the manufacturer’s instructions.

### Molecular detection by RT-qPCR

CCHFV detection was performed using RT-qPCR targeting the L segment of the viral genome, following the protocol of Balinandi [20] with minor modifications. Each 25 µL reaction contained 12.5 µL of 2 × reaction mix (SuperScript™ IV One-Step RT-PCR kit, Thermo Fisher Scientific, USA, 0.5 µL of enzyme mix (2 U/µL; corresponding to 1 U per reaction), 400 nM of each primer, 200 nM of probe, and 5 µL of RNA template.

Amplifications were carried out on a QuantStudio 3 thermocycler (Thermo Fisher Scientific, USA) under the following conditions: 50 °C for 30 min (reverse transcription), 95 °C for 2 min (enzyme activation), followed by 40 cycles of 95 °C for 15 s and 60 °C for 1 min. Fluorescence data were acquired at the end of each 60 °C annealing/extension step. Samples with cycle threshold (Ct) values below 30 were considered positive. Each run included a synthetic CCHFV RNA as a positive control and RNase-free water as a negative control. Positive samples were stored at − 20 °C for downstream analyses.

### Whole-genome sequencing (MinION Mk1C, Oxford Nanopore Technologies)

Samples that tested positive for CCHFV by RT-qPCR were selected for whole-genome sequencing based on Ct values (Ct < 30). RNA extracts were reverse-transcribed into cDNA using the SuperScript™ IV kit (Thermo Fisher Scientific, USA) with random hexamer primers in a 50 µL reaction volume (10 µL of RNA template). PCR amplification was carried out using the VWR MM1 kit (1 ×) (VWR®, Taq DNA polymerase master mix, VWR, France), with 5 µL of cDNA per reaction.

Several primer pairs (Table 1) targeting the S and L genome segments were selected from previously published protocols and laboratory-validated assays. Their performance was evaluated by conventional PCR, and primer pairs producing clear amplicons of the expected size were selected for sequencing. PCR conditions were as follows: initial denaturation at 95 °C for 2 min; 40 cycles of 95 °C for 15 s, 52 °C for 30 s, 72 °C for 40 s; and a final extension at 72 °C for 5 min. Amplicons were visualized on 1% agarose gels, and those showing clear bands were selected for sequencing.

**Table 1:** Primer’s system used for CCHFV genome sequencing

Segment	Primer's ID	Primer	System’s name	Lenght
S Segment	GP_CCHF_seg S_1S	TCTCAAAGAAACACGTGCCGC	Couple 1	530
	GP_CCHF_seg S_21S	CTTACGCCCACAGTGTTCTC		
	GP_CCHF_seg S_530R	GACATRACAATYTCRCCAGG		
	GP_CCHF_seg S_452S	GTGTCAATGCAAATACGGCAG	Couple 2	441
	GP_CCHF_seg S_893R	GGTTTGTTATCATGYTGTCRGC		
	GP_CCHF_seg S_778S	GCCTTGCCAAGCTTGCAGAG	Couple 3	291
	GP_CCHF_seg S_1069R	GGGTGCTTAAGAGTGCCTTC		
	GP_CCHF_seg S_1030S	CGAACTGGGGAAACAACCAAG	Couple 4	345
	GP_CCHF_seg S_1375R	AGGTGTTCAGAGGCTACAATG		
	GP_CCHF_seg S_1448S	GTCAAGGGCAATGCCACCAG	Couple 5	258
	GP_CCHF_seg S_1706R	TCTCAAAGATATCGTTGCCGCA		
	GP_CCHF_seg S_1689R	CGCACAGCCCTTTAAGTRTTT		
	CCHF_seg S_111S	TGGACACCTTCACAAACTC	Couple 6	1,029
	CCHF_seg S_1140R	GACAATTCCCTACACC		
L Segment	GP_CCHF_seg L_1S	TCTCAAAGATATCAATCCCCCC	Couple 1	1,488
	GP_CCHF_seg L_1489R	GAGATCAGCTATCTCCCTGTG		
	GP_CCHF_seg L_1259S	GYAACCTAGGAAATGAACTGTTG	Couple 2	1,708
	GP_CCHF_seg L_2967R	CTTTGCGCATWGCCTGTTCC		
	GP_CCHF_seg L_2809S	GTTGTTGGAGCYATAAGTACTC	Couple 3	1,605
	GP_CCHF_seg L_4414R	GGARTGATTTTCAATGTCTTG		
	GP_CCHF_seg L_4298S	AGGAGRCAAGCTGTCCTTGG	Couple 4	1,888
	GP_CCHF_seg L_6178R	CTTTGACARTTCCAGRTGCTG		
	GP_CCHF_seg L_6022S	CTCAACGAGCAACAAGATGAAC	Couple5	1,584
	GP_CCHF_seg L_7606R	CATGYTCAACATGTACTGTTGTC		
	GP_CCHF_seg L_7491S	GYTACAACCATATGGGTCAGG	Couple 6	1,808
	GP_CCHF_seg L_9299R	GCAGGTCTAGACTCAACTATTC		
	GP_CCHF_seg L_9077S	CTCACTGGTTGGACACCTTTC	Couple 7	1,547
	GP_CCHF_seg L_10624R	CTRTCTGTAGAGCAGTCMAC		
	GP_CCHF_seg L_10253S	GTGARACTGAAAGRCAAGTGC	Couple 8	1,958

Amplicons were purified using AMPure magnetic beads and quantified using a Qubit Fluorometer. Further purification was performed with the Monarch PCR & DNA Cleanup Kit (New England Biolabs, USA). Sequencing libraries were prepared with the SQK-NBD114.24 kit (Oxford Nanopore Technologies, UK) according to the manufacturer’s protocol, and sequencing was performed on a MinION Mk1C platform. Signal data were converted into FASTQ files using Dorado software (Oxford Nanopore Technologies), generating both simplex and duplex reads. Reads were demultiplexed, aligned to a reference CCHFV genome using QIAGEN CLC Genomics software, and assembled to reconstruct the complete viral genome. Sequence quality was assessed based on genome coverage, average read length (N50), and Phred quality scores (Q-scores).

### Phylogenetic analysis

Consensus sequences of the S and L genome segments were aligned with reference CCHFV strains retrieved from GenBank. Multiple sequence alignments were performed using ClustalW implemented in MEGA12 (Molecular Evolutionary Genetics Analysis, version 12) [21]. A total of 1,537 nt from the S segment (PX659872) and 11,530 nt from the L segment (PX659871) were included in phylogenetic analyses. Phylogenetic trees were inferred using the Maximum Likelihood method and the General Time Reversible model of nucleotide substitution, with 1,000 bootstrap replicates to assess node support.

### Statistical analysis

Data were compiled and cleaned using Microsoft Excel and analyzed in R software (version 4.4.2). Descriptive statistics (frequencies and percentages) were used to summarize the characteristics of the sampled cattle and collected ticks. The prevalence of CCHFV detection was calculated by pool and by tick species. At the individual level, the minimum infection rate (MIR) and the maximum likelihood estimate (MLE) were computed using the *PoolTestR* package [22]. The MIR was calculated as the ratio of the number of positive pools to the total number of tested specimens, assuming that each positive pool contained only one infected tick [23, 24]. This measure provides a conservative estimate of infection prevalence. The MLE was used as a more accurate estimator that accounts for variation in pool sizes and provides confidence intervals for the true infection rate within the tested population. The parasitic intensity (mean number of ticks per infested animal) was calculated as the total number of ticks divided by the number of infested cattle. Confidence intervals (95% CI) were estimated using bootstrap resampling. Maps showing sampling sites and origins of sampled cattle were constructed using plotted Quantum GIS (QGIS) version 3.40.1

## Results

### Characteristics of cattle and collected ticks

A total of 299 blood-fed adult ticks were collected between March 2023 and November 2024 from 50 cattle belonging to two breeds: Gudali (30/50; 60%) and White Fulani (20/50; 40%) (Tables 2 and 3).

**Table 2:** Distribution of cattle according to breed and locality of origin in the Adamawa and North regions of Cameroon

Host			*n*	%
Cattle		Overall	50	100
	Breed	Gudali	30	60
		White Fulani	20	40
	Origin	Vina (Adamawa)	28	56
		Poli (North)	11	22
		Touboro (North)	8	16
		Rey-Bouba (North)	2	4
		Tchabal (Adamawa)	1	2

*n* number of animal, Adamawa Region (29/50; 58%), North Region (21/50, 42%)

**Table 3:** Sex and pool distribution of tick species collected from cattle in the Adamawa and North regions of Cameroon (March 2023–November 2024)

Tick species	Adult male *n* (%)	Adult female *n* (%)	Tick pools *n* (%)	Total count *n* (%)
Cattle, *n* = 50				
*Rhipicephalus (Boophilus) microplus*	0 (0.0)	106 (100.0)	11 (12.9)	106 (35.5)
*Hyalomma rufipes*	61 (68.5)	28 (31.5)	29 (34.1)	89 (29.8)
*Rhipicephalus lunulatus*	26 (41.3)	37 (58.7)	24 (28.2)	63 (21.1)
*Hyalomma nitidum*	18 (48.6)	19 (51.4)	20 (23.5)	37 (12.4)
*Amblyomma variegatum*	4 (100)	0 (0.0)	1 (1.2)	4 (1.3)
Total	109 (36.5)	190 (63.5)^*^	85 (100)^a^	299 (100)

*n*: number, %: Percentage, ^a^Sex [male: 38 pools (44.7%)/female: 47 pools (55.3%)]

Most sampled cattle originated from the Adamawa Region (29/50; 58%), including Vina (28/50; 56%), and Tchabal (1/50; 2%). In the North Region, 21/50 cattle (42%) were sampled, with Poli (11/50; 22%), Touboro (8/50; 16%), and Rey-Bouba (2/50; 4%) (Table 2). The mean tick burden per infested animal was 6.0 (95% CI 5.22–6.57), representing the parasitic intensity, while the median was 4.5 (range: 3–7.5). Only adult ticks were recovered, with adult females significantly more abundant (*n* = 190; 63.5%) than males (*n* = 109; 36.5%) (*P* < 0.001; Table 3).

**Fig. 2:**
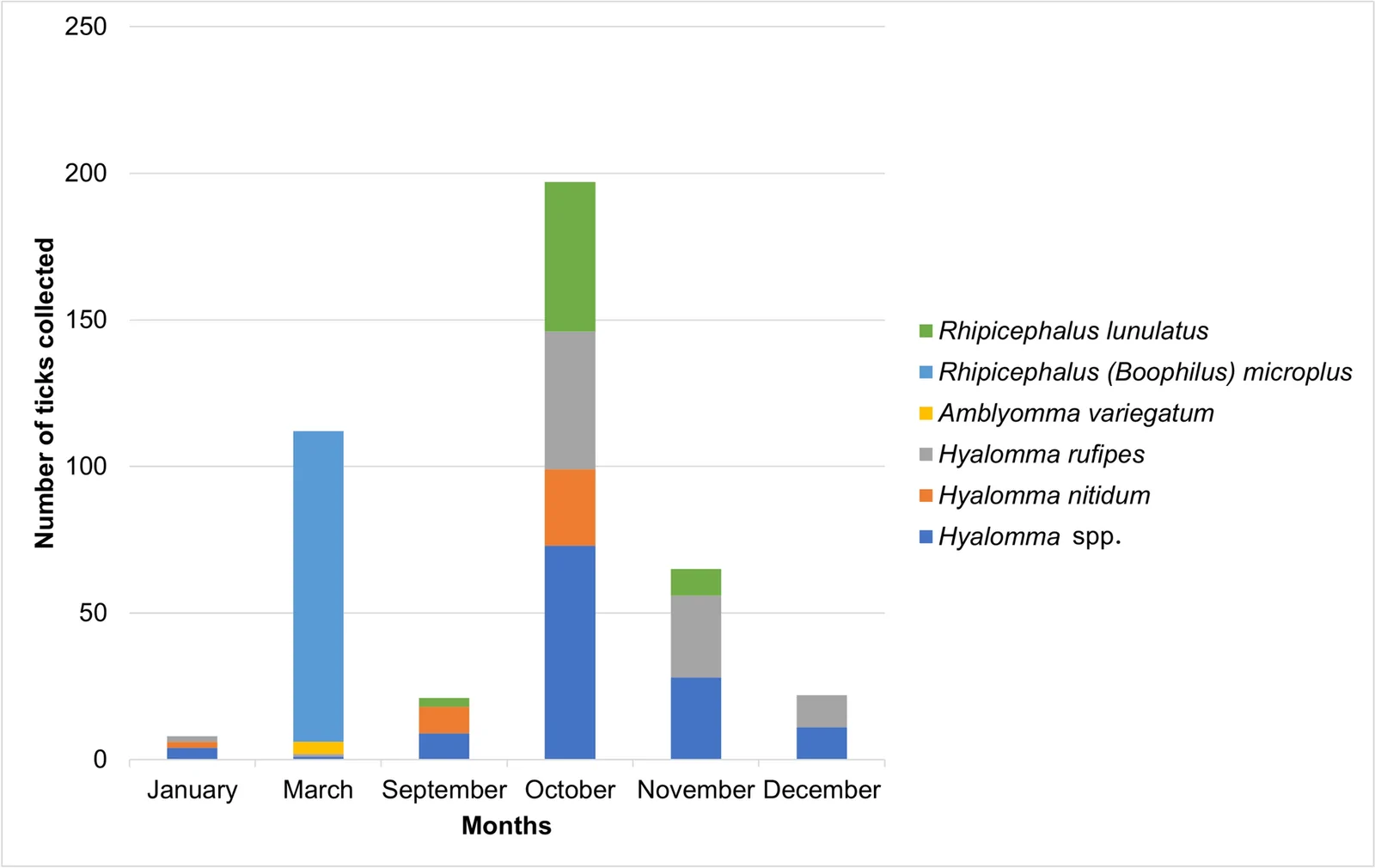
Distribution of blood-fed ticks collected from cattle according to month of collection in the Adamawa and North regions of Cameroon (March 2023–November 2024)

Five tick species were morphologically identified among the 299 ticks collected. Overall, *Rhipicephalus (Boophilus) microplus* was the most abundant species (106/299; 35.5%), followed by *Hyalomma rufipes* (89/299; 29.8%), *Rhipicephalus lunulatus* (63/299; 21.1%), *Hyalomma nitidum* (37/299; 12.4%), and *Amblyomma variegatum* (4/299; 1.3%). Seasonal variation was observed, with *Hyalomma* spp. predominating during the dry season (October), whereas *Rhipicephalus (Boophilus) microplus* was mainly detected during the rainy season (March) (Fig. 2).

Ticks were grouped into 85 pools (1–6 individuals per pool) according to sex, species and host animal. Among these, 47 pools (55.3%) contained 190 females (63.5%) and 38 pools (44.7%) contained 109 males (36.5%) (Table 3).

### Molecular detection of CCHFV

Among the 85 tick pools tested by RT-qPCR assay targeting the L segment of the CCHFV genome, four pools (4.7%) were positive (Ct < 30) (Table 4). The overall MIR was estimated at 1.3% (95% CI 0.4–3.4), with a MLE of 1.4% (95% CI 0.4–3.1). One positive pool was identified as *H. nitidum* (1/20; 5.0% [0.1–24.9]; MIR = 2.4% [0.07–14.2]) and three as *H. rufipes* (3/29; 10.3% [2.2–27.4]; MIR = 3.4% [0.7–9.5]). No viral RNA was detected in pools of *Rhipicephalus (Boophilus) microplus*, *R. lunulatus*, or *Amblyomma variegatum* (Table 5).

**Table 4:** Features of CCHFV positive pools of *Hyalomma* ticks

Pool number	Date of collection	Ct value	Tick			Cattle	
			No. (sex)	Species	Month of collection	Origin	Breed
11–2	06/11/2023	25	1 (F)	*Hyalomma rufipes*	November	Poli	Gudali
22	06/11/2023	25	4 (M)	*Hyalomma rufipes*	November	Vina	Gudali
24–1	09/10/2023	29	4 (M)	*Hyalomma nitidum*	October	Touboro	White fulani
30–1	06/11/2023	28	1 (F)	*Hyalomma rufipes*	November	Vina	Gudali

F: female, M: male, No.: number of ticks

**Table 5:** Infection rate: by pools and individually

Tick species	Pool infection rate			Individual infection rate			
	No. tick pools (%)	No. positive pools (%)	95% IC	No. tick (%)	MIR (%)	MLE (%)	95% IC
Overall	85 (100)	4 (4.7)	1.3–11.6	299 (100)	4 (1.3)	4 (1.4)	0.4–3.1
*Amblyomma variegatum*	1 (10)	0 (00)		4 (1.3)	0 (00)	0 (00)	
*Rhipicephalus (Boophilus) microplus*	11 (12.9)	0 (00)		106 (35.5)	0 (00)	0 (00)	
*Hyalomma nitidum*	20 (23.5)	1 (5)	0.1–24.9	37 (12.8)	1 (2.4)	2 (2.8)	0.2–11.9
*Hyalomma rufipes*	29 (34.1)	3 (10.3)	2.2–27.4	89 (29.8)	3 (3.4)	2 (3.5)	0.9–8.76
*Rhipicephalus lunulatus*	24 (28.2)	0 (00)		63 (21.1)	0 (00)	0 (00)	

MIR: minimum infection rate, MLE: maximum likelihood estimate, IC: confidence interval, No: number

### Sequencing and phylogenetic analysis

Among the four RT-qPCR positive samples with Ct values < 30 (Table 4), only sample 11–2 (Ct = 25) yielded visible amplicons by conventional PCR for the S and L segments. Reference-based assemblies were performed independently for each genomic segment using the closest matching sequences identified by BLAST analysis. Due to the genetic diversity and potential reassortment of CCHFV segments, different reference strains were selected for the S and L segments to optimize mapping efficiency and assembly accuracy (Table 6).

**Table 6:** Nucleotide percent identity values of the sequenced segments with similar regions from the other virus strains (Source: genbank)

Segments	Accession	Percent identity (%)
S segment	ECH 11–2 CAMEROON SEG S (PX659872)	100
	DQ211650.1	97.6
	KY484036.1	97.2
	PP067050.1	92.8
	PP067055.1	92.7
	DQ211648.1	92.7
	MF287636.1	92.2
	DQ211647.1	92.1
	MF547415.1	92.1
	OQ441063.1	91.9
	DQ211641.1	91.9
	DQ211646.1	91.9
	DQ211642.1	87.0
	AF527810.1	86.9
	DQ211645.1	86.7
	DQ211643.1	85.2
	MH133310.1	85.2
	DQ211644.1	85.2
	MH133295.1	84.9
	DQ211649.1	84.5
	DQ211640.1	82.9
	NC_078226.1	82.0
	PP025038.1	81.9
L segment	ECH 11–2 CAMEROON SEG L (PX659871)	100
	DQ211620.1	95.8
	DQ211622.1	95.6
	DQ211613.1	94.7
	DQ211615.1	94.6
	AY389508.2	94.6
	DQ211614.1	94.5
	MF287638.1	94.4
	PP067062.1	94.4
	PP067064.1	94.5
	MF547417.1	94.2
	OQ441065.1	94.2
	PP025040.1	94.4
	DQ211621.1	92.4
	DQ211618.1	88.8
	MH133312.1	88.8
	DQ211617.1	88.7
	MH133297.1	88.7
	DQ211623.1	88.7
	DQ211616.1	86.2
	AY422208.2	85.9
	DQ211619.1	85.8
	DQ211624.1	83.5
	NC_078225.1	78.2

Sequencing quality parameters: S segment: total number of reads (4,802), coverage (333), depth (91%)/L segment: total number of reads (5,321), coverage (99.69), depth (79%)

Full-length sequencing was performed, and there were some ambiguous nucleotide positions (“N”). For phylogenetic analyses, contiguous consensus sequences were reconstructed for each genomic segment by concatenating the obtained fragments. The S segment consensus sequence comprised 1,542 nt (including five ambiguous positions). The L segment consensus comprised 11,565 nt with 35 ambiguous sites. Maximum likelihood phylogenetic trees were inferred using alignments including only homologous and informative nucleotide positions (1,537 nt for S segment and 11,530 for L). Sites containing ambiguous bases (“N”) were excluded from the analysis.

**Fig. 3:**
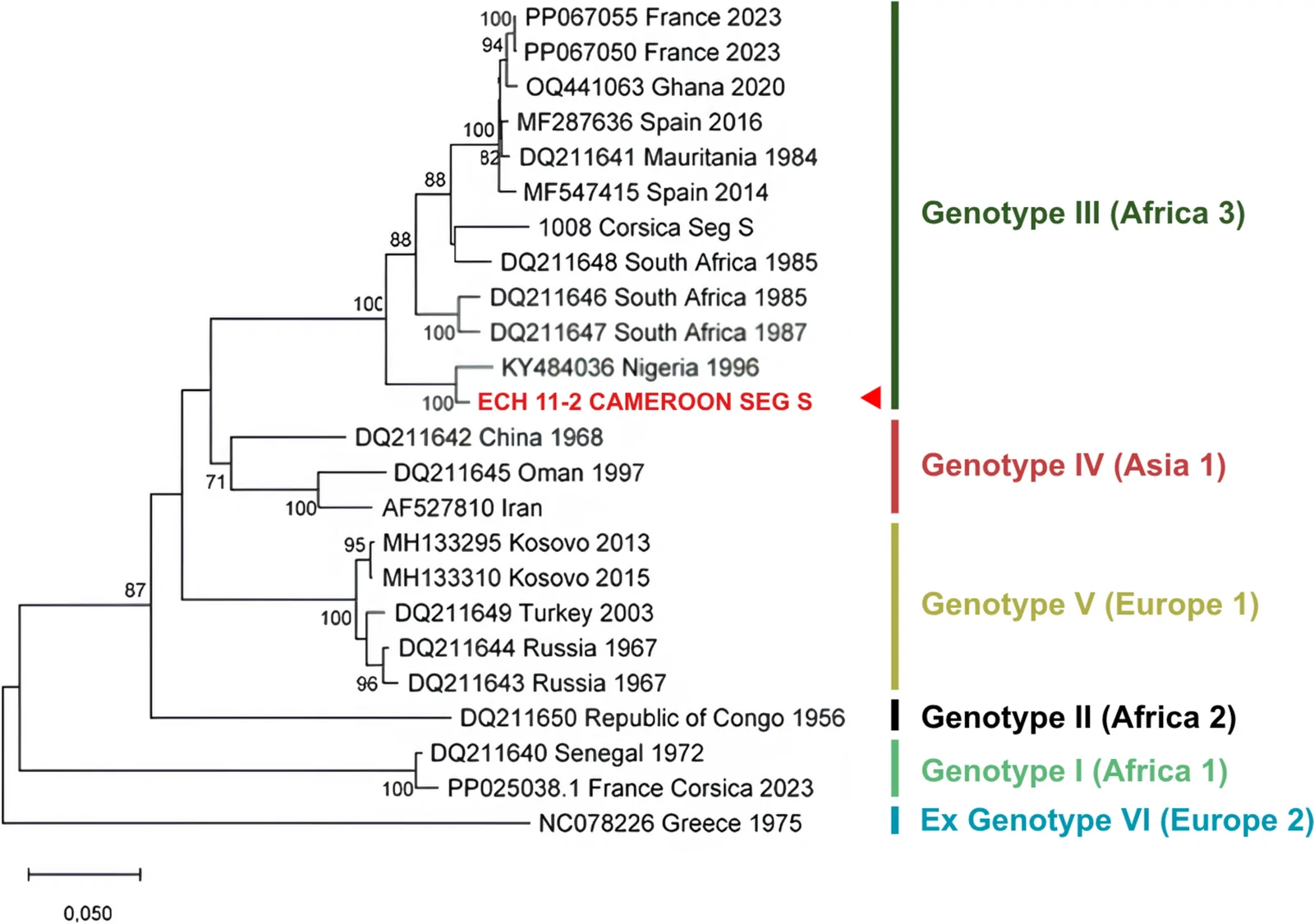
Phylogenetic tree based on 24 partial sequences of the S segment (1,537 nt) of the Crimean-Congo hemorrhagic fever virus (CCHFV). Bootstrap values (1,000 replications) are indicated at the nodes. Genotypes are represented by Roman numerals with their equivalent nomenclature: Africa I (West Africa), Africa II (Central Africa), Africa III (Southern and Western Africa), IV (Middle East/Asia, subdivided into Asia 1 and Asia 2), V (Europe/Turkey), VI (Greece), and VII (Europe). The scale indicates the number of nucleotide substitutions per site. The Cameroon strain is marked with a red triangle

**Fig. 4:**
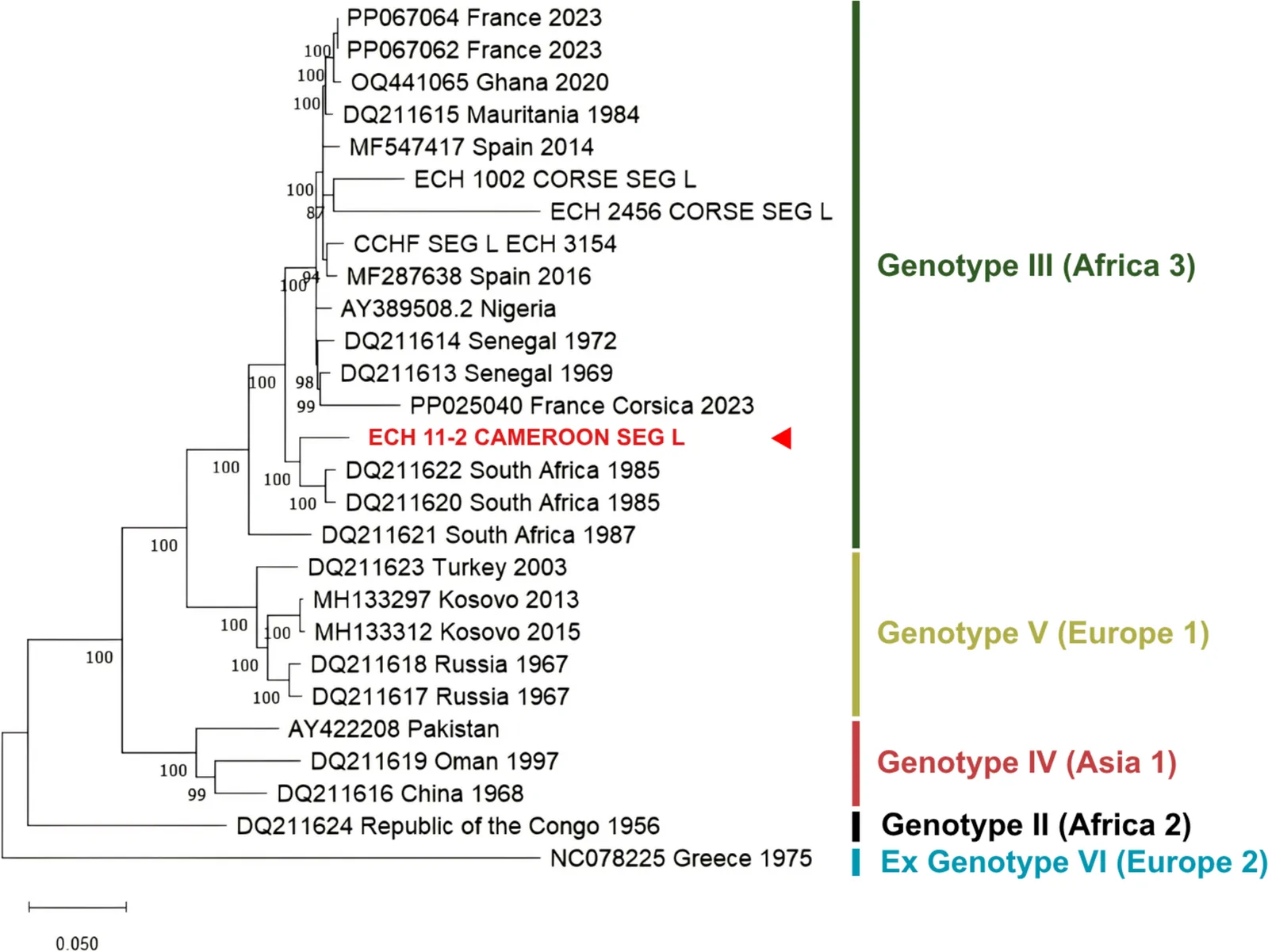
Phylogenetic tree based on 27 partial sequences of the L segment (11,530 nt) of the Crimean-Congo hemorrhagic fever virus (CCHFV). Bootstrap values (1,000 replications) are indicated at the nodes. Genotypes are represented by Roman numerals with their equivalent nomenclature: II (Central Africa), III (Southern and Western Africa), IV (Middle East/Asia, subdivided into Asia 1 and Asia 2), V (Europe/Turkey), and VI (Greece). The scale indicates the number of nucleotide substitutions per site. The Cameroon strain is marked with a red triangle

Phylogenetic analysis of the S segment (Fig. 3) placed sample 11–2 within genotype III (Africa 3), showing high nucleotide identity with strains from Republic of the Congo (DQ211650.1; 97.6%), Nigeria (KY484036; 97.2%) and South Africa (DQ211646; 91.9% and DQ211647; 92.1%). The L segment (Fig. 4) clustered within clade Africa 3 and showed high nucleotide identity with South African strains (DQ211620; 95.8% and DQ211622; 95.6%) (Table 6).

## Discussion

This study presents the first partial genomic characterization of CCHFV from ticks collected from cattle in northern Cameroon. Despite incomplete genome recovery, these data provide valuable insights into CCHFV diversity and circulation patterns in Central Africa, a region that remains underrepresented in global databases [25, 26].

The exclusive detection of CCHFV RNA in *Hyalomma* ticks confirms their role as the primary vectors of the virus in sub-Saharan Africa [27]. The absence of CCHFV RNA detection in *Rhipicephalus* species, despite their higher abundance, supports the hypothesis of *Hyalomma* vector specificity, likely driven by virus–tick biological compatibility [28, 29]. Similar observations have been reported in Mauritania and Nigeria, where *Hyalomma* species are consistently associated with animal exposition to CCHFV [30, 31].

Beyond genetic characterization, the ecological dynamics of tick populations observed in this study provide additional insights into the transmission patterns of CCHFV in the region. The composition and activity of the tick population collected in the present study revealed clear seasonal dynamics with *Hyalomma* species (*H. rufipes* and *H. nitidum)* predominantly observed during the dry season, while *Rhipicephalus (Boophilus) microplus* was more abundant during the rainy season. These patterns are relatively consistent with the Sudanian-Guinean climatic cycle and previous observations across West and North Africa [16, 32, 33]. The seasonal shift between *Rhipicephalus* and *Hyalomma* species highlights ecological specialization linked to rainfall and temperature, conditions that shape tick distribution and host exposure. The seasonal increase in *Hyalomma* populations toward the end of the year (dry season) suggests that October to December represents a period of increased risk for CCHFV transmission. Targeted vector surveillance and control measures during these months could therefore be particularly effective in reducing transmission risk.

The predominance of females observed in *Rhipicephalus* spp. compared to *Hyalomma* spp. is consistent with their differences in feeding behavior and their attachment dynamics on the host. In monoxenous ticks such as *Rhipicephalus (Boophilus) microplus*, females remain attached for long periods to complete engorgement before oviposition increasing their likelihood of being detected during host inspection. In contrast males feed intermittently and move more actively between feeding sites [34]. *Hyalomma* species, which are two or three-host ticks, exhibit different attachment and mating behaviors that may result in a less pronounced sex bias in field collections [35]. The peak observed in October in our study likely reflects post-rainy season conditions in the Sudanian–Sahelian zone, when increased humidity and vegetation favor adult questing activity and enhance host–tick contact. Similar seasonal patterns have been widely reported in West and Central Africa [36, 37].

Phylogenetic analysis of the S and L genomic segments demonstrated that the Cameroonian strain belongs to genotype III (Africa 3), showing high similarity with strains previously reported in Central and Southern Africa [8]. This finding supports the hypothesis of a widespread circulation of the Africa 3 lineage across the region, likely facilitated by livestock movements and transboundary trade [38, 39].

Several limitations should be acknowledged. First, RNA integrity was not formally assessed prior to analysis, and prolonged storage of ticks in ethanol, combined with sample transport, may have affected RNA quality and reduced sequencing efficiency [18]. Second, sequencing was successful for only one sample, likely due to variability in viral load and RNA degradation. Third, the use of pooled samples limits precise estimation of individual infection rates, although MIR and MLE provide reasonable approximations. Results were found in pooled ticks, therefore multiple ticks might have been positive. Finally, the absence of serological data in cattle prevents a comprehensive assessment of host exposure and virus circulation dynamics. Despite these limitations, this study provides robust molecular evidence of active CCHFV circulation in *Hyalomma* tick populations in northern Cameroon; The findings contribute to filling a major gap in genomic data from Central Africa and highlight the importance of integrating entomological and molecular surveillance approaches. Strengthening such One Health strategies will be essential to better understand transmission dynamics and mitigate the risk of zoonotic spillover to humans.

## Conclusions

This study provides the first molecular characterization of CCHFV in *Hyalomma* ticks collected from cattle in Cameroon and contributes to filling a major gap in genomic data from Central Africa. The detection of viral RNA exclusively in *H. rufipes* and *H. nitidum* confirms their role as key vectors in the region. Phylogenetic analysis indicates that the S and L segments are within genotype Africa 3. These findings contribute to filling a major gap in CCHFV genomic data from Central Africa and provide new insights into viral diversity and circulation patterns in the region. Strengthening integrated surveillance approaches combining entomological, molecular, and epidemiological data within a One Health framework will be essential to improve early detection and reduce the risk of human infection.

## Data Availability

Sequences generated in this study are available in Genbank: PX659871 and PX659872.

## Abbreviations

BLAST: Basic local alignment search tool
CCHF: Crimean-Congo hemorrhagic fever
CCHFV: Crimean-Congo hemorrhagic fever virus
cDNA: Complementary deoxyribonucleic acid
Ct: Cycle threshold
CI: Confidence interval
IRD: Institut de Recherche pour le Développement
IRBA: Institut de Recherche Biomédicale des Armées
Inserm: Institut National de la Santé et de la Recherche Médicale
L: Large
MEGA: Molecular evolutionary genetics analysis
MEM: Minimum essential medium
MIR: Minimum infection rate
MLE: Maximum likelihood estimate
nt: Nucleotide(s)
PCR: Polymerase chain reaction
QGIS: Quantum geographic information system
RNA: Ribonucleic acid
RT-qPCR: Reverse transcription quantitative polymerase chain reaction
S: Small
UVE: Unité des virus émergents
